# Double-barreled defense: dual *ent*-miltiradiene synthases in most rice cultivars

**DOI:** 10.1007/s42994-024-00167-3

**Published:** 2024-05-20

**Authors:** Yiling Feng, Tristan Weers, Reuben J. Peters

**Affiliations:** Roy J. Carver Department of Biochemistry, Biophysics and Molecular Biology, Iowa State University, Ames, IA 50011 USA

**Keywords:** Phytoalexins, Diterpenoids, Diterpene synthases, Tandem gene duplication, Neofunctionalization

## Abstract

Rice (*Oryza sativa*) produces numerous diterpenoid phytoalexins that are important in defense against pathogens. Surprisingly, despite extensive previous investigations, a major group of such phytoalexins, the abietoryzins, were only recently reported. These aromatic abietanes are presumably derived from *ent*-miltiradiene, but such biosynthetic capacity has not yet been reported in *O. sativa*. While wild rice has been reported to contain such an enzyme, specifically *ent*-kaurene synthase-like 10 (KSL10), the only characterized ortholog from *O. sativa* (*OsKSL10*), specifically from the well-studied cultivar (cv.) Nipponbare, instead has been shown to make *ent*-sandaracopimaradiene, precursor to the oryzalexins. Notably, in many other cultivars, *OsKSL10* is accompanied by a tandem duplicate, termed here *OsKSL14*. Biochemical characterization of OsKLS14 from cv. Kitaake demonstrates that this produces the expected abietoryzin precursor *ent*-miltiradiene. Strikingly, phylogenetic analysis of *OsKSL10* across the rice pan-genome reveals that from cv. Nipponbare is an outlier, whereas the alleles from most other cultivars group with those from wild rice, suggesting that these also might produce *ent*-miltiradiene. Indeed, OsKSL10 from cv. Kitaake exhibits such activity as well, consistent with its production of abietoryzins but not oryzalexins. Similarly consistent with these results is the lack of abietoryzin production by cv. Nipponbare. Although their equivalent product outcome might suggest redundancy, *OsKSL10* and *OsKSL14* were observed to exhibit distinct expression patterns, indicating such differences may underlie retention of these duplicated genes. Regardless, the results reported here clarify abietoryzin biosynthesis and provide insight into the evolution of rice diterpenoid phytoalexins.

**Supplementary Information:**

The online version contains supplementary material available at 10.1007/s42994-024-00167-3.

## Introduction

Phytoalexins serve an important role in plant defense against pathogens (Ahuja et al. 2012). Their activity is of agricultural importance in crop plants, where such natural products have been intensively studied. As a critically important staple food crop, the arsenal of rice phytoalexins have been extensively investigated for many decades, with the first identified in the 1970s (Valletta et al. 2023). Despite these extended studies, a new group of rice phytoalexins were recently reported, with these abietoryzins further found in a large fraction of the investigated cultivars (Kariya et al. 2023).

Just as with most of the previously known rice phytoalexins (Murphy and Zerbe 2020), the abietoryzins are labdane-related diterpenoids. The biosynthesis of this super-family is initiated by class II diterpene cyclases (Peters 2010). These catalyze bicyclization of the general diterpenoid precursor geranylgeranyl diphosphate (GGPP), prototypically producing the eponymous labdadienyl/copalyl diphosphate (CPP), leading to designation of such enzymes as CPP synthases (CPSs). All land plants must contain a CPS that produces *ent*-CPP, as well as a subsequently acting class I diterpene synthase that yields *ent*-kaurene, for phytohormone biosynthesis (Wang et al. 2023). In angiosperms, this CPS and kaurene synthase (KS) have often given rise to expanded gene families mediating more specialized metabolism (Zi et al. 2014), with members of the latter typically termed KS-like (KSL).

Rice (*O. sativa*) contains two CPSs producing *ent*-CPP, with *OsCPS1* required for gibberellin phytohormone biosynthesis (Sakamoto et al. 2004), while *OsCPS2* is associated with more specialized metabolism (Otomo et al. 2004b; Prisic et al. 2004; Lu et al. 2018). There are then several rice KSLs that act on *ent*-CPP and produce distinct hydrocarbon backbones that characterize the previously known families of labdane-related diterpenoid phytoalexins (Toyomasu et al. 2020). In particular, OsKSL6 yields the *ent*-isokaurene precursor to the oryzalides and related phytoalexins (Kanno et al. 2006), OsKSL7 produces the *ent*-cassadiene precursor to the phytocassanes (Cho et al. 2004), and OsKSL10 yields the *ent*-sandaracopimaradiene precursor to the oryzalexins (Otomo et al. 2004a; Xu et al. 2007a).

**Fig. 1:**
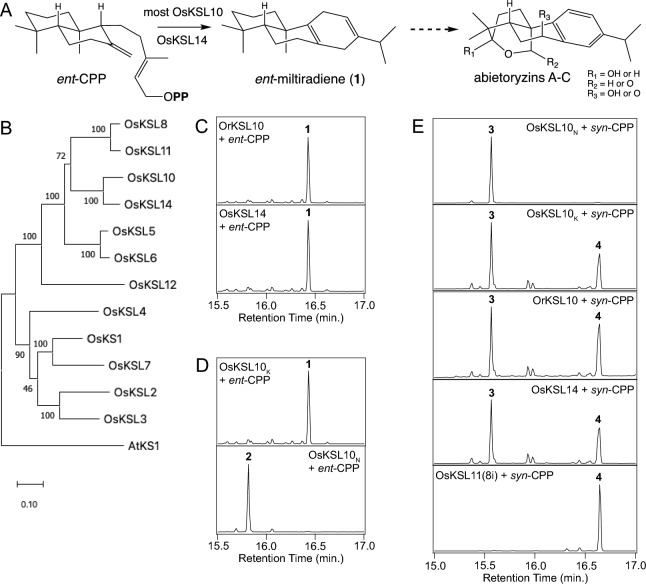
Determination of roles for *OsKSL14* and (most) *OsKSL10* in abietoryzin biosynthesis. **A** Scheme depicting putative role of *ent*-miltiradiene (**1**) in abietoryzin biosynthesis. **B** *OsKSL14* is closely related to *OsKSL10* as indicated by phylogenetic analysis of the rice *KSL* family (tree focused on cv. Kitaake). **C** Production of *ent*-miltiradiene (**1**) from *ent*-CPP by OsKSL14 as indicated by comparison to known activity of OrKSL10 via GC–MS (total ion count (TIC) chromatograms indicating analogous retention time, RT = 16.42 min., and with accompanying mass spectra in Supplemental Fig. S1). **D** OsKSL10_K_ also acts as an *ent*-miltiradiene synthase, exhibiting distinct activity from the previously characterized production of *ent*-sandaracopimaradiene (**2**) by OsKSL10_N_ as indicated by GC–MS analysis (TIC chromatograms shown here, indicating distinct RT = 15.82 min., and with accompanying mass spectra in Supplemental Fig. S3). **E** *OsKSL10* and *OsKSL14* further react with *syn*-CPP and produce *syn*-labdatriene (**3**; RT = 15.57 min.) as well as *syn*-stemodene (**4**; RT = 16.64 min.), which is the primary product of OsKSL11(8i) (TIC chromatograms shown here and with accompanying mass spectra in Supplemental Fig. S5)

Notably, none of the previously characterized OsKSLs reacts with *ent*-CPP to yield an abietane, as is required for abietoryzin biosynthesis (Fig. 1A). More specifically, the aromatic nature of the distal ‘C’-ring in this group of labdane-related diterpenoid phytoalexins would be most readily derived from *ent*-miltiradiene (*ent*-abieta-8,12-diene), whose cyclohexa-1,4-diene arrangement in this KSL formed ring leaves it planar and poised for such aromatization (Gao et al. 2009), which can occur spontaneously (Zi and Peters 2013). Intriguingly, it has been reported that *KSL10* from wild rice (*Oryza rufipogon*) encodes an *ent*-miltiradiene synthase (Toyomasu et al. 2016). Here, identification of *ent*-miltiradiene synthases from domesticated rice is reported.

## Results

While much of the previous work on rice KSLs was carried out with cv. Nipponbare, it has been observed that there are some differences in KSL activity between various subspecies and even cultivars (Xu et al. 2007b; Zhao et al. 2023). Given the emerging use of cv. Kitaake as a model system (Kim et al. 2013; Jain et al. 2019), particularly for investigation of labdane-related diterpenoid phytoalexins (Zhang et al. 2021; Li et al. 2022), an inventory of its *KSL* gene family was carried out, which revealed the presence of a new *KSL*. This was found adjacent to *OsKSL10* (OsKit12g133500) and, building on previous numbering of the rice KSL family, was termed *OsKSL14* (OsKit12g133400). Notably, *OsKSL14* was found to be most closely related to *OsKSL10* (Fig. 1B), and their proximity suggests these arose from tandem gene duplication. Consistent with the lack of previous recognition of this gene, *OsKSL14* does not appear to be present in cv. Nipponbare.

The close phylogenetic relationship of *OsKSL14* to *OsKSL10* and, hence, the *KSL10* from wild rice, along with lack of previously identified *ent*-miltiradiene synthases in domesticated rice, immediately led to the hypothesis that OsKSL14 might serve this function. Accordingly, *OsKSL14* was cloned from cv. Kitaake and incorporated into a previously described modular metabolic engineering system, which enables KSL co-expression in *E. coli* that also produce CPP of defined stereochemical configuration (Cyr et al. 2007). Indeed, comparison of OsKSL14 with a previously described wild rice (*O. rufipogon*) OrKSL10 (Toyomasu et al. 2016) demonstrated that it also reacts with *ent*-CPP to produce *ent*-miltiradiene (Fig. 1C and Supplemental Fig. S1).

As the abietoryzins were reported to be widespread in rice (Kariya et al. 2023), a previously reported phylogenetically representative set of domesticated rice (Zhou et al. 2020), along with a broader set to include other species across the *Oryza* genus (Stein et al. 2018), were examined for the presence of *OsKSL14*. Consistent with a role in such prevalent biosynthesis, *KSL14* appears to be present in all cultivars of *O. sativa* other than Nipponbare (although potentially as a partial pseudo-gene in one other case), as well as all species from the AA-genome clade of *Oryza*, but not in the BB-genome representative *Oryza punctata*. Such presence–absence variation analysis of *KSL10* indicates this neighboring gene is similarly prevalent in *O. sativa*, although it also appears to be a partial pseudo-gene in three cultivars. There is only one cultivar (subspecies indica cv. Larha Mugad) where both seem to be missing. Notably, *KSL10* also appears to be limited to the AA-genome clade of *Oryza*, as it is not present in *O. punctata*.

Intriguingly, while phylogenetic analysis of *OsKSL14* generally matches subspecies (ssp.) assignment, that of *OsKSL10* exhibited some conflict (Supplemental Fig. S2). Specifically, matching the accepted relationships within the *Oryza* genus (Wing et al. 2018), for *OsKSL14* the alleles from cultivars within ssp. japonica subspecies, along with the associated minor ssp. basmati, group separately from those from the other major ssp. (indica) group and associated minor ssp. aus. By contrast, for *OsKSL10*, the allele from cv. Nipponbare is an outlier, with the other alleles more closely related to *OrKSL10*, suggesting that the previously reported activity of the Nipponbare allele (*OsKSL10*_*N*_) might be divergent.

To investigate the potentially more general *ent*-miltiradiene synthase activity of OsKSL10, the allele from cv. Kitaake (*OsKSL10*_*K*_) was cloned and its activity compared to that of OsKSL10_N_ using the modular metabolic engineering system in *E. coli*. Indeed, while OsKSL10_N_ reacts with *ent*-CPP to produce *ent*-sandaracopimaradiene as previously reported (Xu et al. 2007a), OsKSL10_K_ was found to produce *ent*-miltiradiene instead (Fig. 1D and Supplemental Fig. S3). While clearly orthologous, OsKSL10_N_ and OsKSL10_K_ share less than 97% sequence identity at the amino acid level (Supplemental Fig. S4), and their numerous differences preclude ready identification of the particular residue(s) responsible for their distinct product outcomes.

Given that OsKSL10_N_ has been reported to also react with *syn*-CPP to produce *syn*-labdatriene (Morrone et al. 2011), OsKSL10_K_ and OrKSL10 as well as OsKSL14 were similarly examined. Intriguingly, each of these was found to also react with *syn*-CPP. However, these yield not only *syn*-labdatriene but also *syn*-stemodene, which is the major product of OsKSL11 (Morrone et al. 2006), now known to be the allele of OsKSL8 specific to ssp. indica (Toyomasu et al. 2016) and more accurately termed OsKSL8i (Fig. 1E and Supplemental Fig. S5).

Given the inducible nature of the abietoryzins (Kariya et al. 2023), the expression of *OsKSL10* and *OsKSL14* in response to MeJA in cv. Kitaake was analyzed via RT-qPCR. Although only *OsKSL10* and not *OsKSL14* mRNA was detectable prior to induction, consistent with roles in abietoryzin biosynthesis both exhibited increases in transcript levels following treatment with MeJA, with the highest expression found 24 h post-induction (Supplemental Fig. S6).

The biochemical results indicate that cv. Kitaake should produce abietoryzins but cannot make oryzalexins, while cv. Nipponbare should exhibit the opposite metabolite profile, which would be consistent with the previously reported lack of abietoryzins in this cultivar (Kariya et al. 2023). Indeed, targeted analysis of induced seedlings verifies that cv. Nipponbare produces at least oryzalexin C while cv. Kitaake does not, and suggests the opposite is true for the abietoryzins—i.e., at least some may be made by cv. Kitaake, although only after induction, but none by cv. Nipponbare (Supplemental Fig. S7).

## Discussion

Consistent with the previously reported prevalence of abietoryzins (Kariya et al. 2023), here identification of not just a single but dual *ent*-miltiradiene synthases (OsKSL10 and OsKSL14) in most rice cultivars is reported. However, there are certain cultivars that lack any such activity, not least cv. Nipponbare, which does not contain OsKSL14 and whose copy of OsKSL10 exhibits divergent activity in producing *ent*-sandaracopimadiene instead, consistent with the previously reported lack of abietoryzins in this cultivar (Kariya et al. 2023). By contrast, cv. Kitaake seems to produce abeitoryzins but not orzyalexins, consistent with its lack of an *ent*-sandaracopimaradiene synthase. Given the apparently typical biochemical redundancy exhibited by OsKSL10 and OsKSL14, it is unclear what drove the spread of the gene duplication leading to these tandem KSLs. Regardless, the prevalent *ent*-miltiradiene synthase activity encoded by *OsKSL10* and/or *OsKSL14* suggests that biosynthesis of the only recently discovered abietoryzins arose early in evolution of the AA-genome clade of the *Oryza* genus and highlights the need for further investigation of these intriguing natural products.

## Materials and methods

Unless otherwise stated all chemicals were obtained from Fisher Scientific. *KSL* sequences were obtained via BLAST searches with the known genes from cv. Nipponbare against the Gramene *Oryza* database (Tello-Ruiz et al. 2022). *OsKSL14* and *OsKSL10*_*K*_ were cloned from cv. Kitaake, while *OsKSL10*_*N*_ from Nipponbare is that previously described (Xu et al. 2007a), and a gene (optimized for expression in *Escherichia coli*) was synthesized for the previously described *OrKSL10* (Toyomasu et al. 2016). All genes were truncated to remove the N-terminal plastid targeting sequence and characterized via a previously described modular metabolic engineering system (Cyr et al. 2007). The resulting diterpene products were extracted and analyzed by gas chromatography with mass spectrometry (GC–MS) as previously described (Jia et al. 2022), with the addition of passage over silica gel to remove confounding polar metabolites from the extract prior to GC–MS analysis. Given the presence of putative splicing errors in the predicted coding sequences, genomic sequences were obtained for *KSL10* and *KSL14* and iteratively aligned by BLAST with the exons from the known examples. An error in the predicted junction between exons 10 and 11 in *OsKSL10* was found by comparison of that predicted and cloned for cv. Kitaake, and the correction (shift of four nucleotides from exon 10 to 11) applied in this re-analysis. Similarly, frame-shifting mutations were also noted in the *KSL10s* predicted by this re-analysis for *Oryza glumaepatula* and *Oryza glaberrima*, so those originally found in Gramene were used instead. These coding sequences were used for the presented phylogenetic analyses, which were carried out using MEGA11 (Tamura et al. 2021). Gene expression levels were analyzed by reverse transcription quantitative polymerase chain reaction (RT-qPCR) using the primers listed in the supplemental information (Table S1), and 10-day (post-germination) cv. Kitaake seedlings induced with methyl jasmonate (MeJA) as previously described (Wang et al. 2011). For diterpenoid measurement, rice seedlings were grown, induced and extracted as described by Kariya et al. (2023), with analysis by liquid chromatography with mass spectrometry (LC–MS/MS) as previously described (Zhang et al. 2021), and the relevant diterpenoids identified using an oryzalexin C standard and the relative retention times and MS/MS detection reported by Kariya et al. (2023) for the abietoryzins.

## Supplementary Information

Below is the link to the electronic supplementary material.

Supplementary file1 (PDF 1226 KB)

## Data Availability

All data generated or analyzed in this study are available from the corresponding author upon reasonable request.
