# Supplementary material for: Double-barreled defense: dual ent-miltiradiene synthases in most rice cultivars
Source: aBIOTECH. 2024 May 20;5(3):375–80. doi: 10.1007/s42994-024-00167-3 (PMC11399519; doi:10.1007/s42994-024-00167-3)
Supplement: Supplementary file 1 — Supplementary file1 (PDF 1226 KB) [file 42994_2024_167_MOESM1_ESM.pdf]

<u>**Supporting Information for**</u>**:**

Double-barreled defense: Dual *ent*-miltiradiene synthases in most rice cultivars

Yiling Feng, Tristan Weers and Reuben J. Peters*

Roy J. Carver Department of Biochemistry, Biophysics & Molecular Biology, Iowa State University, Ames, IA 50011, USA

Figure S1

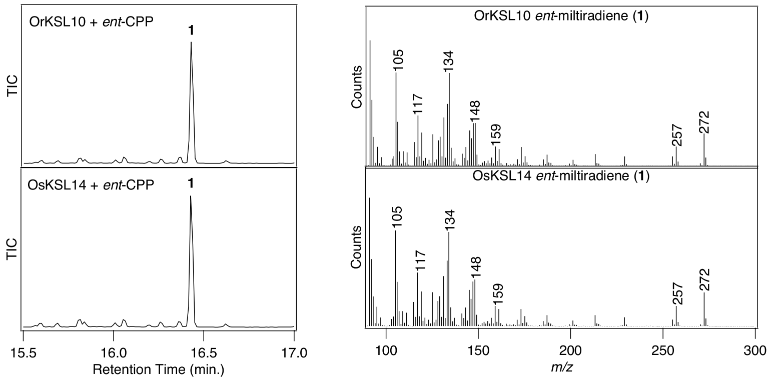

Fig. S1: Verification of OsKSL14 *ent*-miltiradiene synthase activity. GC-MS total ion count (TIC) chromatograms and mass spectra (MS) for *ent*-miltiradiene (**1**) produced from *ent*-CPP by OsKSL14, as identified by comparison of retention time (RT = 16.43 min.) and MS to known activity of OrKSL10.

Figure S2

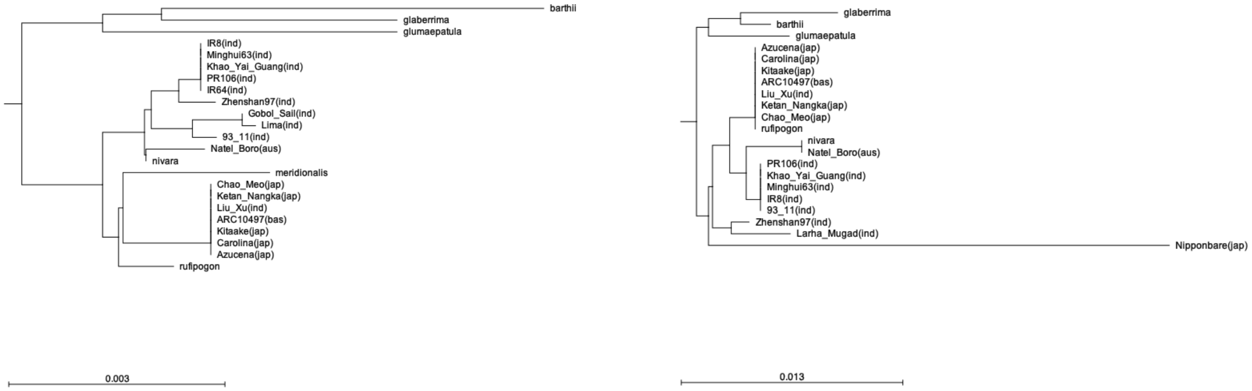

Fig. S2: Phylogenetic trees for *KSL14* (left) and B) *KSL10* (right), based on alignment of coding sequences. Distinct species of *Oryza* indicated by lowercase names (i.e., *nivara*, *rufipogon*, *barthii*, *glaberrima*, *meridionalis* and *glumaepatula*), while capitalized indicates cultivar names with sub-species indicated in parentheses (i.e., japonica, jap; indica, ind; basmati, bas; Australasian; aus). The *O. barthii* and *O. glaberrima* and *O. glumaepatula* sequences were designated as the outgroup in both trees.

Figure S3

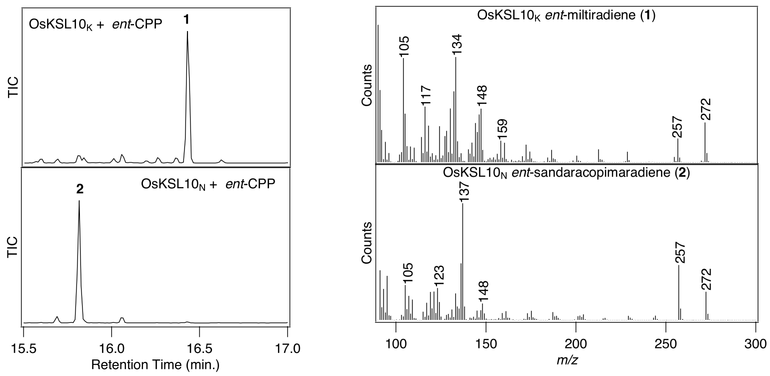

Fig. S3: Verification of OsKSL10$_K$ *ent*-miltiradiene synthase activity. GC-MS TIC chromatograms and MS demonstrating distinct production of *ent*-miltiradiene (**1**) from *ent*-CPP by OsKSL10$_K$, again identified by comparison of RT (16.43 min.) and MS to known activity of OrKSL10 (c.f., Fig. S1), as well as contrast to the known production of *ent*-sandaracopimaradiene (**2**), RT = 15.66 min. and distinct MS, by OsKSL10$_N$.

Figure S4

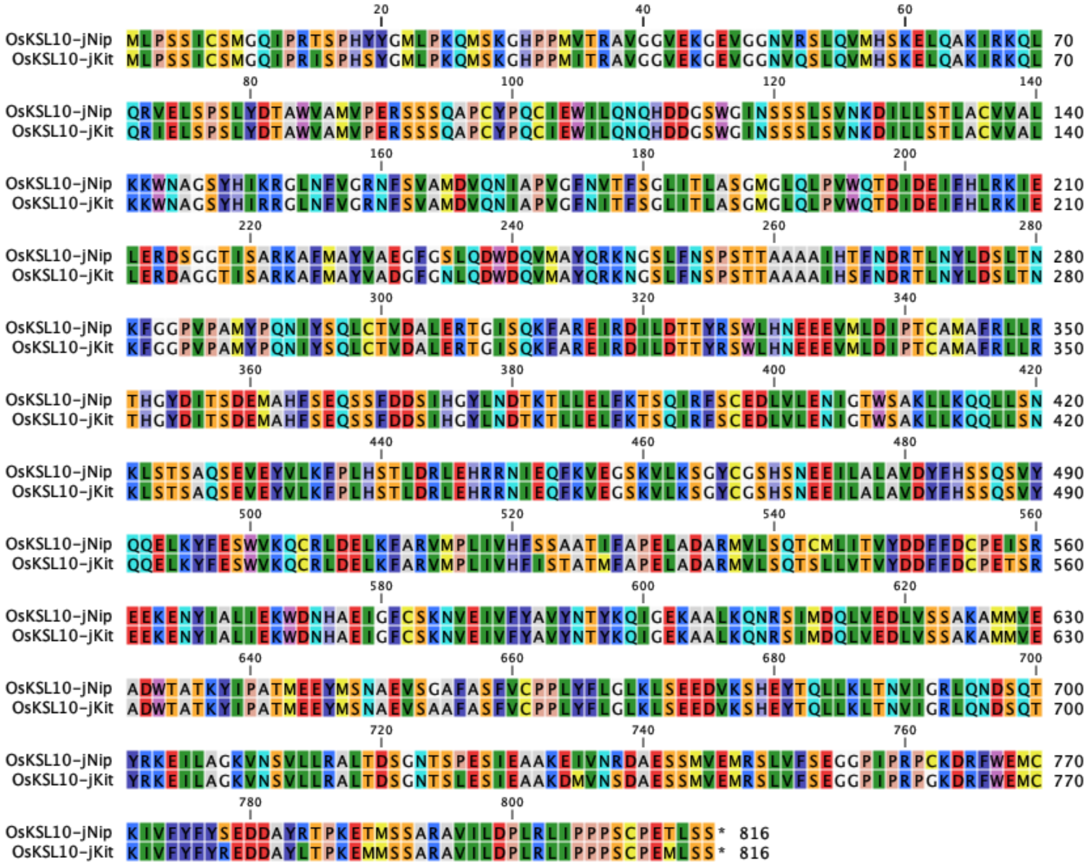

Fig. S4: Amino acid alignment of OsKSL10$_N$ and OsKSL10$_K$.

Figure S5

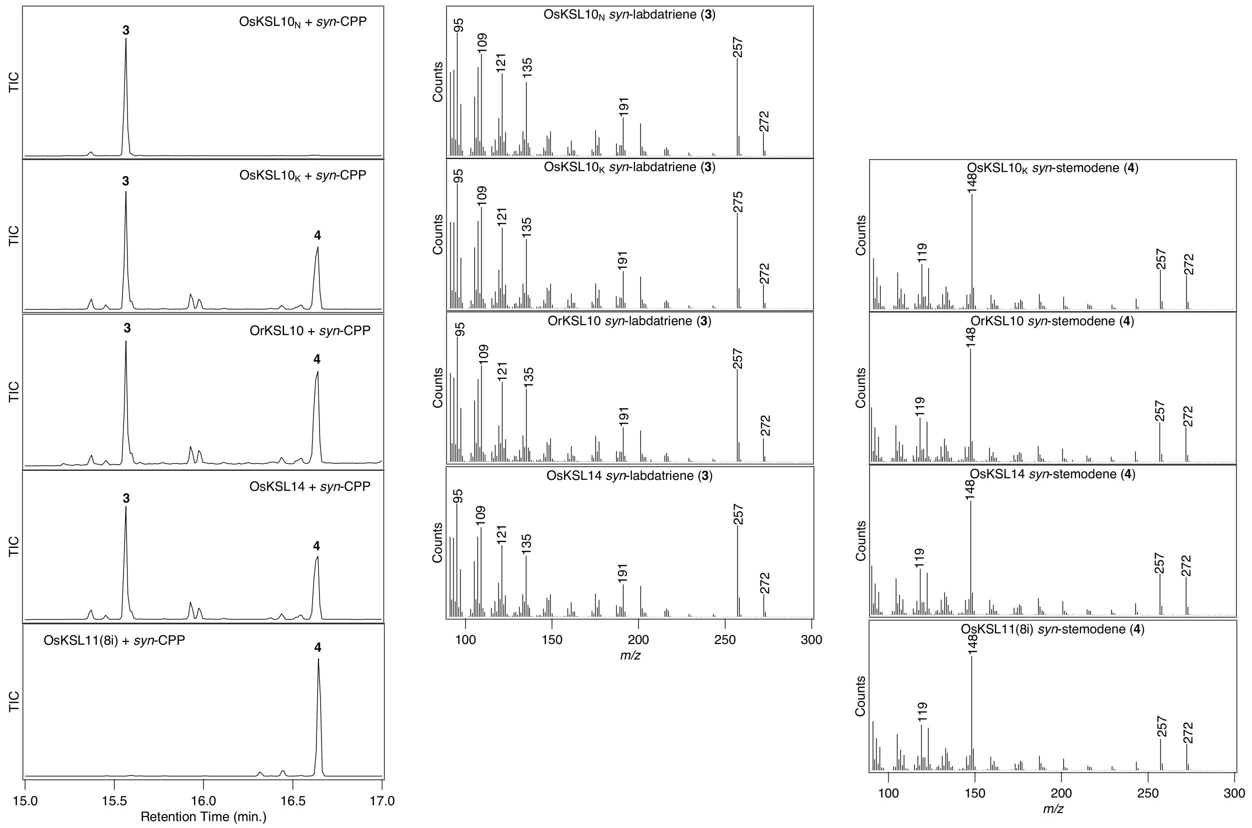

Fig. S5: Verification of reactivity with *syn*-CPP. GC-MS TIC chromatograms and MS demonstrating production of *syn*-labdatriene (**3**), again identified by comparison of RT (15.57 min.) and MS to known activity of $OsKSL10_N$, but also *syn*-stemodene (**4**), RT = 16.64 min., as similarly identified by comparison to OsKSL11(8i), by $OsKSL10_K$, OsKSL10 and OsKSL14.

Figure S6

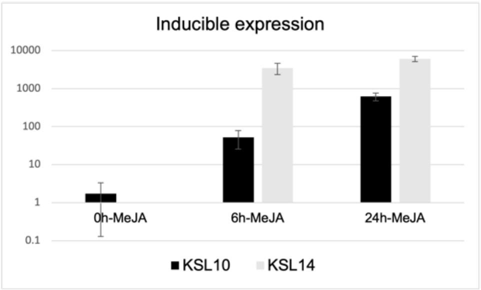

Fig. S6: Expression level of *OsKSL10* and *OsKSL14* in response to induction by MeJA as measured by RT-qPCR analysis of cv. Kitaake seedlings.

Figure S7

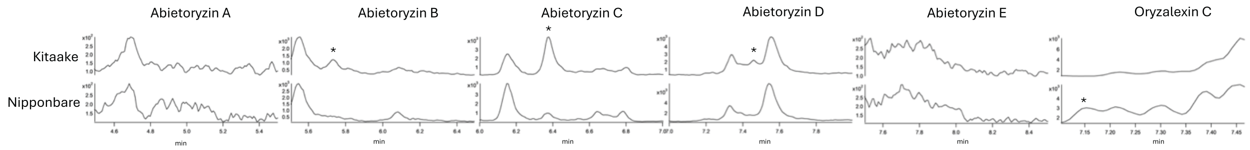

Fig. S7: Comparison of diterpenoid profiles for cv. Kitaake and cv. Nipponbare. Shown are indicated MS/MS chromatograms from LC-MS/MS analysis of control and induced seedlings.

Supplemental Table S1: Primers used for RT-qPCR analysis.

ACT1	CTCAGCACATTCCAGCAGAT	ACAGATAGGCCGGTTGAAAA
OsKSL10$_K$	AGTTCGCAAGAGTGATGCCA	AGTTACCAGGAGGCTGGTCT
OsKSL14	GCCTCCTGAATGACTCCCAG	TGACTCGGCGAAACACTTGA
